# Endoscopic transpapillary gallbladder drainage for localized acute cholecystitis confined by segmental adenomyomatosis using a novel rotatable sphincterotome

**DOI:** 10.1055/a-2721-9273

**Published:** 2025-11-05

**Authors:** Yoshihiro Goda, Kuniyasu Irie, Yuto Matsuoka, Tomomi Hamaguchi, Hideyuki Anan, Yoshimasa Suzuki, Shin Maeda

**Affiliations:** 1 218758 Gastroenterology, Yokohama City University Hospital, Yokohama, Japan; 2 Gastroenterology Division, Yokohama City University, School of Medicine, Yokohama, Japan

Endoscopic transpapillary gallbladder drainage (ETGBD) for localized acute cholecystitis in the fundal region confined by a segmental adenomyomatosis (ADM)-related stricture is technically challenging due to the difficulty of guidewire passage through the stricture. When the catheter tip is not directed toward the stricture, guidewire passage becomes more difficult. A novel rotatable and flexible sphincterotome (Engetsu; Kaneka Medix, Osaka, Japan; Fig. 1 ) allows the fine adjustment of the tip orientation 1 2 3 , thereby facilitating selective guidewire insertion 4 . Here, we present a case in which the Engetsu catheter enabled successful guidewire passage through a segmental ADM-related stricture, resulting in successful ETGBD ( Video 1 ).

**Fig. 1:**
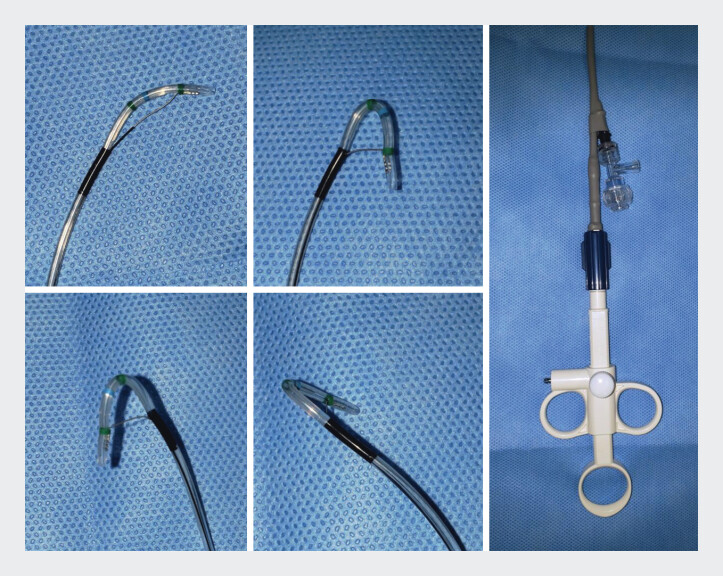
A novel sphincterotome, Engetsu (Kaneka Medix, Osaka, Japan), with smooth rotatability allowing the fine adjustment of the catheter tip orientation.

A novel rotatable sphincterotome was useful for performing ETGBD in a case of localized acute cholecystitis in the fundal region confined by segmental adenomyomatosis.Video 1

A 65-year-old man with a distal biliary obstruction ( Fig. 2 ) caused by neuroendocrine carcinoma developed localized acute cholecystitis. Computed tomography revealed a ventrally located ADM-related stricture in the gallbladder body, with inflammation localized to the fundus ( Fig. 3 ). Percutaneous transhepatic gallbladder drainage was contraindicated due to the absence of hepatic contact, and endoscopic ultrasound-guided gallbladder drainage was considered difficult because of insufficient gallbladder distension ( Fig. 3 **c** ). Therefore, ETGBD was selected. Cholangiography from the gallbladder neck revealed a stricture in the body caused by ADM. Using a conventional catheter, the device could not be directed toward the stricture, resulting in failed guidewire passage ( Fig. 4 **a** ). The catheter was then exchanged for the Engetsu catheter. Based on computed tomography findings ( Fig. 3 **a, b** ), the tip was oriented ventrally by bending and rotating the handle, enabling alignment with the stricture. The guidewire was successfully advanced through the ADM-related stricture ( Fig. 4 **b** ), and a 6 Fr endoscopic nasobiliary drainage tube was placed in the gallbladder fundus ( Fig. 4 **c** ). The inflammation resolved without any adverse events, and the patient was discharged following replacement with a plastic stent.

**Fig. 2:**
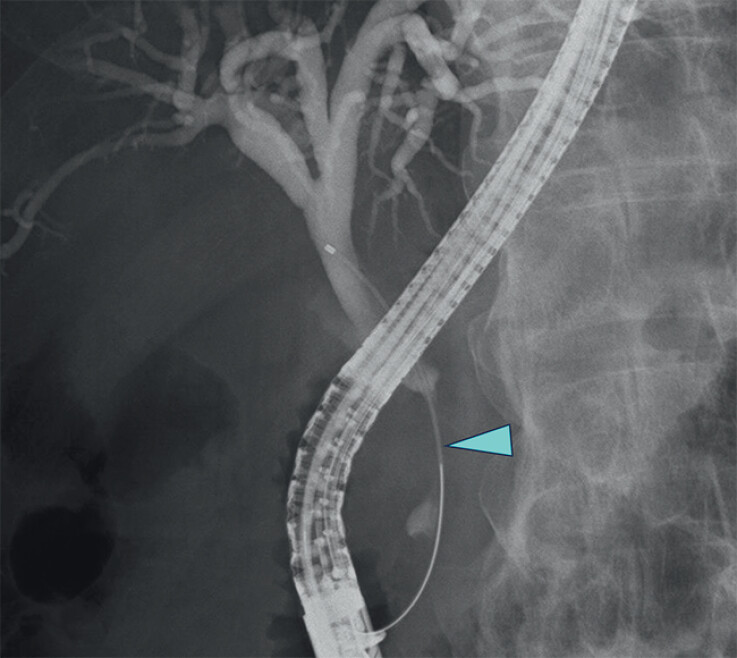
Cholangiography showing a distal biliary obstruction caused by neuroendocrine carcinoma (arrowhead).

**Fig. 3:**
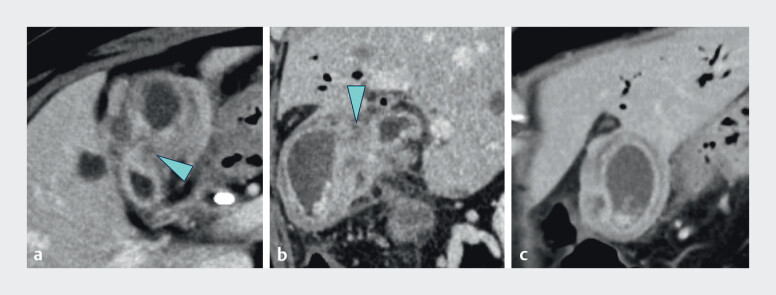
Computed tomography showing a ventrally located stricture in the gallbladder body due to adenomyomatosis (arrowhead) and localized cholecystitis confined to the fundus: **a** axial image, **b** sagital image and **c** coronal image.

**Fig. 4:**
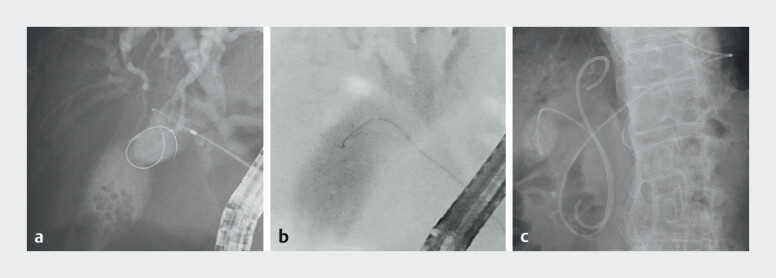
**a** Using a conventional catheter, the device could not be directed toward the stricture, resulting in failed guidewire passage. **b** Adjustment of the Engetsu catheter orientation enabled successful guidewire advancement through the segmental stricture. **c** A 6 Fr endoscopic nasobiliary drainage tube was successfully placed.

To the best of our knowledge, this is the first report of ETGBD for localized acute cholecystitis confined by a segmental ADM-related stricture in which the flexible bending and rotational capabilities of the Engetsu catheter enabled successful drainage.

Endoscopy_UCTN_Code_TTT_1AR_2AK

## Notes

Endoscopy E-Videos https://eref.thieme.de/e-videos
*E-Videos* is an open access online section of the journal *Endoscopy* , reporting on interesting cases and new techniques in gastroenterological endoscopy. All papers include a high-quality video and are published with a Creative Commons CC-BY license. Endoscopy E-Videos qualify for HINARI discounts and waivers and eligibility is automatically checked during the submission process. We grant 100% waivers to articles whose corresponding authors are based in Group A countries and 50% waivers to those who are based in Group B countries as classified by Research4Life (see: https://www.research4life.org/access/eligibility/ ). This section has its own submission website at https://mc.manuscriptcentral.com/e-videos .
